# Safety Assessment of Sedation Services in Radiology at Shaukat Khanum Memorial Cancer Hospital and Research Center, Peshawar

**DOI:** 10.12669/pjms.40.8.9326

**Published:** 2024-09

**Authors:** Faraz Mansoor

**Affiliations:** 1 Faraz Mansoor, MBBS, FCPS (Anesthesia), FCPS (critical care medicine), Fellowship in critical care medicine, Consultant Anesthetist, Shaukat Khanum Memorial Cancer Hospital and Research Center, Peshawar, Pakistan Email: mansoor_faraz@hotmail.com

**Keywords:** Radiology, Sedation, Patient Safety

## Abstract

**Objective:**

The purpose of this retrospective observational study was to evaluate the safety of newly started sedation services in the department of Radiology Shaukat Khanum Memorial Cancer Hospital and Research Center Peshawar, Pakistan.

**Methods:**

Medical record of the patients who underwent sedation for different types of imaging studies at Shaukat Khanum Memorial Cancer Hospital, Peshawar center were retrospectively reviewed to evaluate the safety outcome of sedation. Process variables included preoperative assessment, obtaining informed consent acquisition, confirmation of nulla per os (NPO) status, monitoring during the procedure, post-anesthetic care unit (PACU) handover, and discharge instructions. The monitored complications included instances of cardiac arrest, respiratory arrest, hypoxia, apnea and vomiting.

**Results:**

No major adverse events, such as respiratory or cardiovascular arrest, were observed among the patients in our study. A limited number of cases experienced minor events, specifically hypoxia and apnea, all of which were effectively addressed through simple maneuvers. However, one patient with previously undiagnosed obstructive sleep apnea encountered upper airway obstruction, necessitating the discontinuation of the procedure. Subsequently, all patients were safely discharged to their homes from the recovery area.

**Conclusion:**

Sedation in Radiology department can be safely performed by adhering to a structured approach aligned with hospital policies and procedures.

## INTRODUCTION

A substantial number of cancer patients undergoes diagnostic and interventional procedures at the Radiology Department of Shaukat Khanum Memorial Cancer Hospital and Research Center (SKMCH). Given the presence of underlying malignancies and co-morbidities, these patients are at increased risk of deterioration when exposed to sedatives and anesthetic agents, stemming from compromised physiological reserves due to chemotherapy or malnutrition, and sometimes airway obstruction can result from upper airway tumor. However, there is not enough data available from Pakistan.

The department of Anesthesiology at Shaukat Khanum Memorial Cancer Hospital and Research Center in Peshawar devised a structured program specifically tailored for patients requiring sedation and anesthesia outside the traditional operating theater setting.^1^-^3^ This program was implemented in April 2021 concurrent with the introduction of new operation theaters and anesthesia services at our Peshawar center. Undoubtedly, this structured approach contributes to a safer environment for patients.^4^,^5^ Notably, this patient population is susceptible to respiratory and cardiovascular events.^6^ Therefore, the main objective of this retrospective study was to evaluate the safety of the patients undergoing sedation in Radiology department for different procedures. Top of Form

## METHODS

This retrospective observational study was conducted at Shaukat Khanum Memorial Cancer Hospital and Research Center (SKMCH) in Peshawar during the timeframe of April to September 2022, with the requisite study approval obtained from the institutional review board (IRB). (Exemption certificate) Anesthesia department started providing sedation to patients undergoing different diagnostic procedures in SKMCH, Peshawar in April 2022. We retrospectively analyzed the medical record of a total of one hundred and ten patients, ranging from 1-72 years old who underwent various imaging studies, including CT, MR imaging, PET scan, and DTPA scanning. All patients requiring sedation in Radiology were included in our study cohort. However, those with airway difficulty, missing data, and those undergoing bronchoscopy and endoscopy were excluded. Informed consent was waived as this was a retrospective observational study.

### Anesthetic technique

A meticulous pre-procedure assessment was conducted by the Anesthesia consultant for all patients, and the recorded information was documented in the Hospital Information System using a specially designed anesthesia preoperative evaluation template. The anesthesia evaluation encompassed a focused history and physical examination, with integral components such as ASA status and airway assessment performed in all cases. Special attention was devoted to pediatric patients, considering the high risk associated with upper and lower airway infections or congestion, during the sedation procedures. High risk patients were identified and a sedation plan formulated, and patient education was provided to all the adult patients and to parents/guardians of children including clear instructions about the intake of food, water and milk. Patients concerns were also addressed during these visits. In addition to this all patients received a telephone call one day before the procedure to ensure that they had followed the pre-procedure instructions and they arrived on time on the day of procedure.

On the day of the procedure, an informed and written consent is taken explaining all the benefits and risks of the sedation and the alternative options available. It was also ensured that the patients had followed the nil per os instructions. Clear fluids were allowed until two hours before the procedure. Vitals signs were checked by the nursing staff and documented in the record.

The three most frequently employed intravenous drugs were Propofol, Midazolam and Ketamine. Propofol was given in a dose of 1-2 mg/kg initially followed by small doses as needed to keep the patient sedated. Ketamine was used in a dose of 0.5-1 mg/kg via intravenous route and Midazolam 0.05mg/kg. Inhalational Sevoflurane was utilized in children with challenging intravenous access. Pain relief for patients was managed with Paracetamol, NSAIDs, and opioids as needed, while the availability of the reversal agents such as Naloxone and Flumazenil was ensured for emergency situations.

In cases of difficult intravenous access, ultrasound-guided intravenous access was employed. Pre-induction vital signs were assessed and recorded in the anesthesia notes for all patients, and continuous monitoring during the sedation procedure included ECG, pulse oximetry, respiratory rate, non-invasive blood pressure and end-tidal CO2 with records documented at five minutes intervals in anesthesia notes. Anesthesia consultant remained present with all the patients during the sedation procedure.

Following the successful completion of the procedure, patients were transferred to the recovery area for observation and monitoring of the complications. During their transport from the radiology department to the PACU, adequate monitoring including heart rate, blood pressure, pulse oximetry, respiratory rate, and level of consciousness, was maintained. The patient transfer personnel also ensured the availability of necessary emergency drugs and airway equipment during the patient transport from Radiology to the PACU area that they carry in a dedicated bag, which is located on the third floor of the hospital.

Upon arrival at the PACU, a detailed handover was provided to the recovery area team. Patients were continuously monitored in the PACU and discharged upon meeting the discharge criteria, assessed using the Post-anesthesia Discharge Scoring System (PADS). Clear verbal and written discharge instructions, along with emergency contact numbers, were provided to all patients, parents, or guardians at the time of discharge.

### Definition and outcomes:

### Cardiac Arrest:

Requirement of cardiopulmonary resuscitation

### Hypoxia:

Sustained pulse oximetry saturation <90% for greater than 1 minute duration.

### Respiratory arrest:

Apnoea for greater than 1 minute.

### Apnoea:

Temporary suspension of breathing for greater than 20 seconds.

### Data collection

Data was retrospectively collected from the electronic medical record and anesthesia notes, and entered on hardcopies, and latter saved in Microsoft Excel spreadsheet.

## RESULTS

Between April and September 2022, diagnostic radiological procedures were performed on a cohort of 110 patients that included both adult and pediatric populations. Among the age group 78 patients (70.91%) were < 18 years old and 32 patients (29.09%) were adults. Descriptive characteristics of the patients and the type of complications are shown in Table-I. MRI was the most common indication for sedation which was followed by the CT scan. Sedation procedure couldn’t be completed in one patient (0.009%). Overall, adverse events occurred in 12 (10.90%) patients, including: 3 (2.7%) episodes of apnoea, 3 (2.7%) episodes of vomiting and 6 (5.45%) episodes of hypoxia. No major adverse events were recorded, for example, cardiac arrest or respiratory arrest.

**Table-I:** Descriptive characteristics of patients and complications.

Characteristics		Numbers/%
Total number of Patients		110
Gender	Male	64 (58.18%)
	Female	46 (41.81%)
Age	<18 year	78(70.91%)
	>18 year	32 (29.09%)
Type of Procedures	CT Scan	47 (42.72%)
	MRI	49 (44.54%)
	PET Scan	10 (9.09)
	DTPA Scan	4 (3.63%)
Complications	Cardiac Arrest	0
	Respiratory Arrest	0
	Apnoea	3 (2.72%)
	Vomiting	3 (2.72%)
	Hypoxia	6 (5.45%)

Hypoxia was successfully managed by re-positioning of the airways and increasing the oxygen concentration. Similarly apneic episodes just required bag and mask ventilation. Post-discharge, none of the patients required readmission to the hospital due to sedation-related complications, such as vomiting. Additionally, one patient was referred to our Lahore center due to an anticipated difficult airway, as an MRI-compatible anesthesia machine was not available at that time.

## DISCUSSION

The decision to undertake this retrospective study was taken as our hospital started new operation theatres and provision of non-operating room anesthesia/sedation services at SKM Peshawar. The results of this study shows that most of the patients can be safely provided sedation without major adverse effects and brief periods of deranged physiology were successfully managed with simple maneuvers. Most of the patients requiring sedation services in the radiology were children (<18 year). Before the start of this sedation program, the SKM leadership ensured that it is according to the standards set by the Joint Commission International.

Hospital policies and procedures provide basic recommendations which are based on the current scientific evidence. Francis report highlights the importance of standards, training, regular audits and organization.^7^ The goals of sedation as stated by the American Society of Anesthesiologists are very clearly stated and in order to achieve these goals, our institute has developed a structured approach which include: written guidelines to be followed by all the team members involved in the provision of care: a program of quality assurance to regularly monitor the practices by doing audits: and ongoing education at the departmental level.^1^ These guidelines cover all the aspects of patient care including preanesthesia assessment, identification of high-risk patients, informed consent, use of safety checklist, monitoring, patient transport, recovery area and written instructions are provided at the time of discharge.^4^,^5^

The set of results described in this retrospective study are similar to those documented by the previous studies. Hoffman et al’s data provide direct evidence that application of the American Society of Anesthesiologists process model can reduce the risk of procedural sedation.^8^ Egelhoff et al have also established the safety and efficacy of sedation in hospital radiology department by using a structured approach.^9^ These guidelines not only help in developing policies and procedures for the hospitals but can also help in identifying high-risk patients.

Patients with head and neck tumors can pose challenges to the anesthetists due to airway related problem. For example, radiotherapy to the head and neck region can result in jaw stiffness and difficulty in mouth opening.^10^ Computerized tomography or magnetic resonance imaging, and awake nasal endoscopy are very helpful during the pre-anesthesia assessment of patients with head and neck tumors.

What are the pertinent clinical implications arising from the outcomes observed in this study? These findings suggest that the hospitals should develop policies and procedures based on strong evidence or recommendations which will help reduce the patient harm.

### Limitations of the study

The principal limitation of this study pertains to its single-center design, focusing predominantly on a study population comprised largely of cancer patients.

### Conclusion and recommendation

The implementation of a structured sedation program has facilitated the delivery of sedation services characterized by enhanced safety and quality which is attributed to adherence to international guidelines and standards of care. The author recommends for the standardization of the sedation practices on a national level in Pakistan.
